# Association between cannabis use and complications related to ulcerative colitis in hospitalized patients: A propensity matched retrospective cohort study: Erratum

**DOI:** 10.1097/MD.0000000000017046

**Published:** 2019-08-30

In the article, “Association between cannabis use and complications related to ulcerative colitis in hospitalized patients: A propensity matched retrospective cohort study”,^[1]^ which appears in Volume 98, Issue 32 of *Medicine*, Dr. C. Roberto Simons-Linares appears incorrectly as Simons-Linares C. Roberto.
